# The Interplay of Biomimetics and Biomechatronics

**DOI:** 10.3390/biomimetics7030096

**Published:** 2022-07-21

**Authors:** Hartmut Witte

**Affiliations:** Group of Biomechatronics, Faculty of Mechanical Engineering, Technische Universität Ilmenau, Max-Planck-Ring 12, D-98693 Ilmenau, Germany; hartmut.witte@tu-ilmenau.de

**Keywords:** biomimetics, biomechatronics, biology, engineering, interdisciplinarity, education

## Abstract

Biomechatronics is an engineering subject in which biomimetics as a method is one of its two supporting pillars: biology for engineering, or Bio4Eng. This is contrasted with biocompatible design, or Eng4Bio, examples of which are human-serving systems, such as exoskeletons, and biomedical engineering. The paper aims to illustrate that the research fields of biomimetics, biomechatronics, and biomedical engineering are not in competition but mutually supportive. The current attempts to place biomechatronics under the umbrella of biomimetics or biomedical engineering are therefore not expedient; they deprive the subject of its strength of combining Bio4Eng and Eng4Bio at any time in a task-related manner. In addition to research and development, however, the training of the specialists supporting the subjects must not be disregarded and is therefore described based on a proven design.

## 1. Introduction

Beyond the debate whether the subject was initiated with the coining of the terms “biomimicry”, “biomimetics” or “bionics”, the supplementing of technological skills by using knowledge from the observation of nature is an old technique of mankind. Its formal application in science is often attributed to Leonardo da Vinci (1452–1519) and Matthew Baker (1530–1613) [1]. What concerns the definition of “biomimetics”, we refer to DIN-ISO 18458-2016 (Biomimetics—terminology, concepts and methodology) [2] and VDI 6220.1-2021 (Biomimetics—fundamentals, conception, and strategy) [3].

Biomechatronics, on the other hand, is a term that only became possible with the emergence of mechatronics and the release of the trademark claiming the term in 1982. It was successfully introduced by Hugh Herr at M.I.T. as an addition to the use of the term biomimetics for activities in the field between biology and mechatronic technology and has been successfully represented by him internationally for over 30 years, mostly by patents (cp. [4,5,6,7,8,9,10,11,12,13,14,15,16,17,18,19,20,21,22,23,24,25,26,27,28,29,30,31,32,33,34,35,36,37,38,39,40,41,42,43,44,45,46,47,48,49]). Brody, as early as in 2005, addressed biomechatronics to be one of the “10 emerging technologies” [50]. Is biomechatronics to be understood as a subfield of biomimetics? At a time of the rise of mechatronics, the term addressed the interaction of this discipline (rather than the entirety of engineering) with life sciences. From his own concern, Herr started with the aspect of mechatronics for medicine, thereby practically narrowing the term initially to biomechatronics as a subfield of biomedical engineering, and this is how it is still frequently perceived internationally today [51,52,53]. At the time of writing this article, Web of Science points to 6153 publications entitling “biomechatronics” and “biomedical engineering”. However, Herr based his work in medicine on biomimetics—at least a quarter of his publications we cite directly addresses biomimetics, mostly concerning his own work. This fact is widely ignored: there are only 10 findings in Web of Science for “biomimetics” and “biomechatronics”. Consequently, for the benefit of biomimetics, two equally important objectives of the subject biomechatronics have to be named: “engineering for biology” (Eng4Bio; e.g., for human-serving systems and in biomedical engineering) and “biology for engineering” (Bio4Eng, first dominated by biomimetics). Due to the successes in Eng4Bio, Bio4Eng increasingly had to cover aspects of biocompatibility in addition to biomimetics, and therefore, with advancing digitalization, Bio4Eng now also includes aspects of human–machine interaction, especially ergonomics and usability. Since no international standard is available, as a working base we defined biomechatronics in the following way: “Biomechatronics is the development and improvement of mechatronic products and processes using biological and medical knowledge.” [54]. Thus, in contrast to biomimetics, it is primarily anchored in engineering. Since biomechatronics makes use of biomimetics, which is the largest part of Bio4Eng beneath human–machine interaction, it is a sibling of biomimetics, but not restricted to it [55].

Since no internationally accepted definition of biomedical engineering is available, we refer to the widely used definition of Michigan Technological University, “Biomedical engineering is the application of the principles and problem-solving techniques of engineering to biology and medicine. This is evident throughout healthcare, from diagnosis and analysis to treatment and recovery, and has entered the public conscience though the proliferation of implantable medical devices, such as pacemakers and artificial hips, to more futuristic technologies such as stem cell engineering and the 3-D printing of biological organs.” [56], which illustrates that responsibility is claimed for biology and medicine, but applications are directed to human biology with the focus on medicine

**Figure 1:**
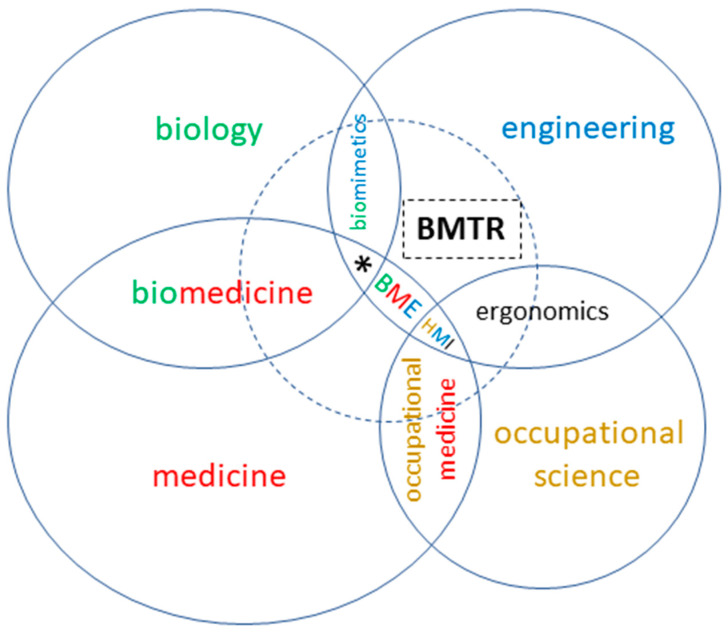
Activities of biomechatronics in relation to the related scientific fields (dashed circle). BMTR: biomechatronics; BME: biomedical engineering; HMI: human–machine interaction; Asterisk *: engineering for biology (“E4B”).

Figure 1 illustrates our perception of the field graphically. Additionally, in order to reliably represent the integrative character of biomechatronics in all of the given fields, the concept of the “biomechatronic system” is helpful [57].

Biomimetics and biomechatronics are therefore two overlapping and thus complementary scientific fields. In the following, some aspects of the exchange and cooperation between the disciplines will be discussed.

## 2. Material and Methods

### 2.1. Problem Description

Recently, the term biomechatronics has become fashionable in general. Both in publications [58,59] and in job advertisements for professorships, there is a reversal of system and system components: “biomechatronics in biomedical engineering”, “biomechatronics in bionics”, and, in extreme detail, “biomechatronics for measurement systems in the biomedical sciences”, illustrating a current trend to cannibalize biomechatronics by other scientific fields. Now, the scientific game of segregation of new from old scientific fields is an established basis of scientific progress (for example, physiology first spun off from anatomy; from the latter physiological chemistry, and from the latter molecular biology), and the appropriation of innovative developments by large disciplines is part of the standard repertoire of academic institutions. However, that is not the point here. The subject matter of a discipline is defined by what its practitioners do: “Biomechatronics is what biomechatronics engineers do.” Furthermore, “pars pro toto” (a part taken for the whole) and “totum pro parte” (the whole taken for a part) are rhetorical figures at first, but they can have huge effects. Building on 20 years of our own academic representation of the subject of biomechatronics in research and teaching, we must point out what a loss the detachment from Herr’s idea of the equal status as well as simultaneity of Bio4Eng and Eng4Bio would be for technical progress. The author is free to argue here: as an engineer and physician with many years of experience in biology (functional morphology), he does not have to take sides with any of the components of biomechatronics; in particular, regarding biomimetics, as a co-founder of the German biomimetics competence network BioKoN, he also may be allowed to make critical statements towards his own community.

### 2.2. The Role of Biomimetics in Biomechatronics

In engineering science, we consider biomimetics quite pragmatically as a method, without therefore questioning the scientific claim of the subject so named. In practice, it is rather normal to actively realize the part of technical biology in biomimetics itself together with biologists, because for which technical requirements do biologists have prepared biological principles at hand in the sense of “technological pull”? The way of the “biology push” is more probable and thus often the implementation of a biological insight for the development of products. In this context, one must be aware of the fact that technical advancement in biomimetics is strongly dependent on the current topics in the life sciences, and those partly are driven by the available analysis technologies (which nowadays often are embodied by biomechatronic systems).

For the expansion of the “technology pull” approach, consideration of the “soft skills” component is indispensable. Biologists, physicians, and engineers are trained differently, learn different ways of thinking, as well as have different methods and terminologies. Most future professionals may still vacillate between biology and medicine as a course of study at school, but the decision will rarely be between life or engineering sciences. We are all shaped since childhood with respect to our preferences for science. Thus, working effectively in truly interdisciplinary fields, such as biomimetics and biomechatronics, requires a willingness to engage with other subjects, and the mindsets of the people practicing them, in more than a superficial way. In biomimetics, for all the initial enthusiasm for interdisciplinary collaboration, the honeymoon phase easily turns into that of resignation if this insight is missing. Therefore, life and engineering scientists need to be actively introduced to each other and to each other’s ways of thinking and terminologies. Acquiring this empowerment is a core element of higher education biomimetics.

### 2.3. Differences between Biomimetics and Biomechatronics

In biomechatronics, the problem is twofold: potential partners for technical developments come primarily from biology and medicine—two major subjects, two technical languages. It is therefore clear that biomechatronics can only be realized in a team, ideally embedded in an environment of biology, medicine, and technology. In education, only a few universities can offer this environment; industry has more diverse opportunities here through the suitable location of company parts.

Figure 1 shows how biomechatronics can be realized under these boundary conditions or (to use a biological term) “environmental” conditions. Due to the indispensability of human-compatible design of products in the product field of human-serving systems, including those for biomedical technology, interaction with occupational sciences is necessary as the fourth pillar of work. Biomechatronics does not live as a niche subject between the other subjects but integrates parts of them in the entire field. The dashed circle in Figure 1 can be compared to the search area of a bee swarm, the main areas of nectar collection change regularly depending on supply and demand. Own daughter colonies with frequent contacts are found close to biology (bionics) and medicine (biomechatronics in biomedical engineering) due to good location factors. The biological metaphors in the description of a technical science may illustrate that biomechatronic engineers by no means claim to be “specialists for everything”, but saprophytically cooperate with specialists from other disciplines.

**Figure 2:**
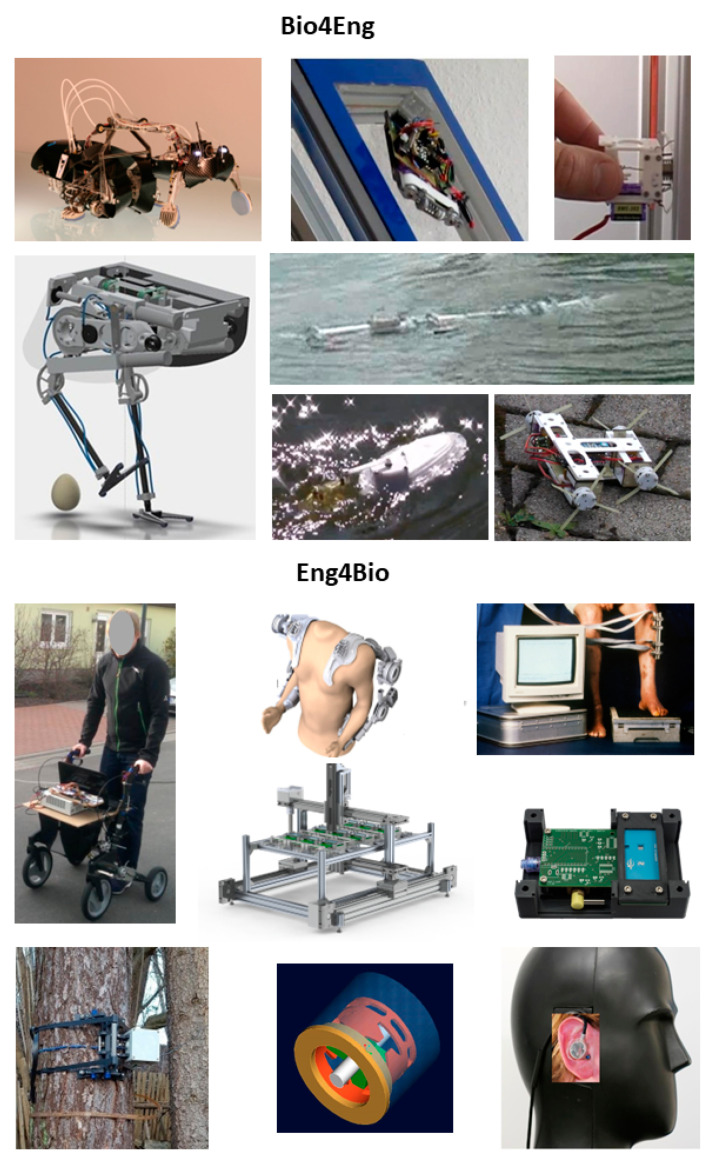
Examples of biomechatronic development. In reading order, please find for Bio4Eng the climbing robot *RatNic* (coop. Tetra GmbH lmenau, granted by the German Ministery of Education and Research BMBF) [60]; the window-cleaning robot *MatBo*t; the adhesive gripper *TaCare*; the bird-like robot *eNandu* (coop. FSU Jena, Gentle Robotics); the swimming robots *CSnake* and *Urmele*; the small wheg robot *T-Whex*. For Eng4Bio: the walker *eRolli*; the modular exoskeleton *Leviaktor* (coop. FSU Jena, HS Aalen, Gottinger, BM innovations, LSK, granted by German Ministery of Education and Research BMBF), the figure is provided by Gottinger; fixateur externe instrument for measurement of fragment motions in DoF 6 (coop. RU Bochum) [61]; the system view and module of the System for Automated Cell Cultivation and Analysis (*SACCA*; granted by the Carl-Zeiss Stiftung); a dendrometer; a sensor for gripping forces DoF 6, 20 × 25 mm [62]; and a Personalized Miniaturized Dosimeter (*PMD*; granted by BGN Mannheim Erfurt) [63].

Examples of our own biomechatronic work are given in Figure 2. “Bio4Eng” provides examples from biorobotics, which for biomimetic realization of function need mechatronics. “Eng4Bio” shows examples of human-serving systems and measurement setups. Due to the present focus of biomedical engineering on the aspects of human biology and medicine, we created a chance to be contacted by biological and biomedical scientists for mechatronic tool building and supporting experiments, and in Figure 1 the corresponding area is marked by an asterisk representing “engineering for biology” (E4B).

## 3. Results

### 3.1. Education in Biomechatronics

For student training in biomechatronics, such examples naturally form the backbone of an engineering course. Since 2002, three training concepts have been implemented. For four years, the diploma course in mechatronics ran over 3 years of specialization, with the clear premise that the graduates would be accepted by industry without restriction as fully fledged engineers. This successful phase was ended by the fact that with the introduction of the “Bologna” ECTS system with bachelor’s and master’s degrees, only 1.5 years were realized for the master’s degree program—too little for a solid education. Consequently, the biomechatronics education started already in the bachelor’s program and extended over another 1.5 years, together again forming 3 years. However, the advantage of longer learning with more intensive penetration of the subject matter proved to be no longer feasible, since part of the audience of the bachelor’s program left the university after graduation, and new students were admitted to the master’s program. The prior knowledge of the students was therefore very different, and part of the propaedeutics from the bachelor’s program had to be repeated in the master’s program. This situation, which was unsatisfactory for all concerned, was remedied by extending the master’s program to two years and increasing the amount of time per year spent on specialization.

**Figure 3:**
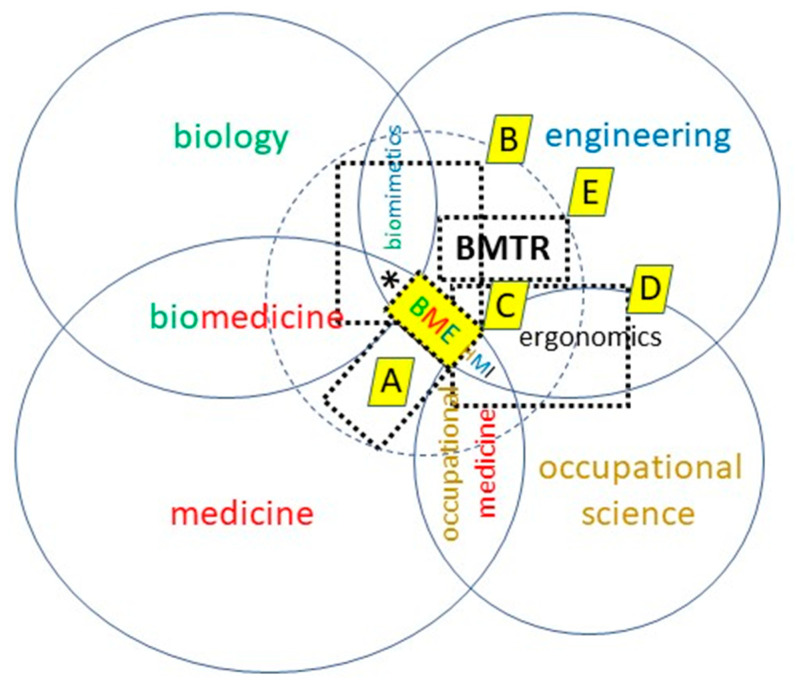
Overlay on Figure 1: Superimposed are the modules of the master’s study programs in which the associated content is taught. “A”—Anatomy and Physiology; “B”—Bio-oriented Methods in Engineering; “C”—Basics of Biomedical Engineering; “D”—Human-Serving Systems; “E”—Biomechatronics. Asterisk * : engineering for biology (“E4B”).

Figure 3 illustrates how biomechatronics now is taught in the master’s program in five modules. Accompanying this, there is a larger module over 1 year for a (bio) mechatronic design project (workload of 480 h).

The basics of medicine are provided in the module “Anatomy and Physiology” (“A” in Figure 3, workload 150 h). Biomimetics is the focus of the module “Bio-oriented Methods in Engineering” (“B”, 150 h). In addition to a smaller theory part (20 h), a biomimetic design project is realized in group work (60 h). At the same time, the students practice working with agile methods. The remaining 70 h of the module are devoted to biomechanics, the methods of which are already used during the biomimetic design project. The learning experience of this project flows back into the biomechatronic design project. The module “Basics of Biomedical Engineering” (“C”, 150 h) is offered by our colleagues in Germany’s oldest (founded in 1954) Institute of Biomedical Engineering and Informatics. The module “Human-Serving Systems” (“D”, 150 h) introduces the basic principles of human–machine interaction. Those are the pre-requisites for the “core” module “Biomechatronics” (“E”, 150 h).

### 3.2. Intensification of Cooperation between Biomimetics and Biomechatronics for Mutual Benefit

Beneath personal interest, the question remains as to what should motivate biologists to contribute to technology. In Germany, biomimetics has experienced a surge in development for about a decade, with massive public funding for joint projects between biology and technology under the label “Bionics”, with an estimated EUR 50 million invested. As a result, biomechatronics has been able to make great progress in biorobotics (cp. Figure 2). In the USA, DARPA promoted biomimetic developments with greater perseverance, and in the People’s Republic of China, investments in this postulated future field are growing steadily. Biologists have been able to conduct basic research on this basis, which could no longer be adequately financed from normal research funding. However, how can the motivation to cooperate with technology be maintained if the extrinsic motivation is removed by funding the objects of intrinsic motivation? For biomechatronics, the answer is simple: biology has great need for experimental setups of the latest technology—the natural partners and suppliers of this technology are biomechatronics engineers. The small gap between biomimetics, biology, and biomedical engineering (asterisk in Figure 1) is surprisingly not yet occupied by any established terminus (cp. [58,59]. Our proposal is “engineering for biology” (quite different from the term biomedical engineering, which at present does not really cover the development of devices for the whole field of life sciences—leaving a wide-open gap). In this field, biomechatronics can offer biology and biomimetics a win–win situation in return for the benefit of mechatronic product development in collaboration.

## 4. Conclusions

Increased collaboration between biomimetics, biomedical engineering, and biomechatronics is of benefit to all involved in the interaction of life sciences and engineering. Mutual appropriation and demarcation cause friction losses and harm the development of all subjects.

## Data Availability

Not applicable.
